# Predictive performance of the waist-to-height ratio and the conicity index for diagnosing excess body fat in children

**DOI:** 10.1590/1984-0462/2025/43/2025114

**Published:** 2025-11-14

**Authors:** Thaís Pessoa Barros, Carla Soraya Costa Maia, Luis Felipe Nunes de Oliveira, Thaynan dos Santos Dias, Eric Wenda Ribeiro Lourenço, Ribanna Aparecida Marques Braga, Juliana Raissa Oliveira Ricarte, Soraia Pinheiro Machado, Maria Dinara de Araújo Nogueira

**Affiliations:** a Universidade Estadual do Ceará, Fortaleza, CE, Brazil.; b Universidade de São Paulo, São Paulo, SP, Brazil.

**Keywords:** Body composition, Pediatrics, Overweight, Obesity, Students, Waist-height ratio, Composição corporal, Pediatria, Sobrepeso, Obesidade, Estudantes, Razão cintura-estatura

## Abstract

**Objective:**

To evaluate the performance of waist-to-height ratio (WHtR) and conicity index (CIndex) as predictors of body fat percentage (BF) in children, as well as to determine the most appropriate cut-off points for this population.

**Methods:**

This school-based study assessed 314 children aged five to nine years. Body composition was evaluated using bioelectrical impedance analysis (BIA), and children were classified as having excess or normal BF according to sex. WHtR and CIndex were calculated using their respective formulas. To assess the predictive power of CIndex and WHtR, the area under the curve (AUC) of Receiver Operating Characteristic was analyzed, using excess BF by BIA as the gold standard.

**Results:**

WHtR was considered a good predictor of excess BF in both sexes, with AUC values greater than 0.8. CIndex was classified as a poor predictor (AUC<0.7 in both sexes). WHtR showed a significantly higher AUC than CIndex (p<0.001), according to the Wald test. Given the higher accuracy of WHtR, the optimal cut-off points to identify excess BF in children were >0.47 for boys and >0.45 for girls.

**Conclusions:**

WHtR demonstrated good performance in predicting excess BF in the children evaluated and may be included in child health surveillance as an indicator of alterations in body composition.

## RESUMO

**Objetivo:**

Avaliar o desempenho da relação cintura-estatura (RCE) e do índice de conicidade (IC) como preditores do percentual de gordura corporal (GC) em crianças, bem como determinar os pontos de corte mais adequados para essa população.

**Métodos:**

Estudo de base escolar que avaliou 314 crianças de cinco a nove anos de idade. A composição corporal foi avaliada por meio de bioimpedância elétrica (BIA), e as crianças foram classificadas com ausência ou presença de excesso de GC de acordo com o sexo. A RCE e o IC foram calculados por meio de suas respectivas fórmulas. Para a avaliação do poder preditivo do IC e RCE, foi analisada a área sob a curva (AUC) da *Receiver Operating Characteristics* utilizando como padrão-ouro o excesso de GC pela BIA.

**Resultados:**

A RCE foi considerada um bom preditor do excesso de GC em ambos os sexos, apresentando AUC maiores que 0,8. O IC foi classificado como um preditor ruim (AUC<0,7 em ambos os sexos). A RCE apresentou AUC significativamente maior do que o IC (p<0,001) de acordo com o teste de Wald. Considerando-se a melhor acurácia da RCE, os pontos de corte ideais para identificar o excesso de GC nas crianças foram: >0,47 para os meninos e >0,45 para as meninas.

**Conclusões:**

A RCE teve um bom desempenho na predição do excesso de GC nas crianças avaliadas, podendo ser incluída na vigilância da saúde infantil como indicador de alterações na composição corporal das crianças.

## INTRODUCTION

 Childhood obesity has shown exponential growth over the past four decades, currently affecting approximately 40 million children and adolescents worldwide. In Brazil, nearly 16 million individuals in this age group are classified as overweight, reflecting the impact of social determinants of health on the population’s health-disease process, including limited access to healthy foods and physical inactivity.^1^ In the Northeast region, data from the Food and Nutrition Surveillance System of the Ministry of Health indicate that the Unified Health System (SUS) serves approximately 453 thousand adolescents with excess weight, with the state of Ceará presenting the highest rate in the region.^2^

 Childhood obesity is directly associated with cardiometabolic risks and its persistence into adulthood, contributing to early morbidity and mortality. Dietary factors, such as the increased consumption of energy-dense and micronutrient-poor foods, early introduction of non-breast milk, as well as other behavioral determinants, directly increase the risk of obesity in children. From this perspective, continuous monitoring of the nutritional status of this population is essential, particularly regarding body fat.^3-5^

 Advanced methods such as bioelectrical impedance analysis (BIA) and dual-energy X-ray absorptiometry (DEXA) offer high precision in assessing body composition and can detect excessive adiposity, enabling the identification of potential alterations associated with cardiometabolic outcomes. However, their high cost and the lack of standardized reference values for pediatric use limit their application in clinical practice. Alternatively, anthropometric indicators such as the waist-to-height ratio (WHtR) and the conicity index (CIndex) have emerged as more accessible and feasible options, especially in resource-constrained settings.^6,7^

 The CIndex is an anthropometric indicator developed to estimate central body fat distribution. This index has been widely used as a relevant marker of cardiovascular and metabolic risk, given the strong association between abdominal obesity and adverse health outcomes.^8^ The RCE is another anthropometric indicator that has gained prominence in recent years as an important screening tool and measure of abdominal adiposity and prediction of excess weight in all age groups. Additionally, this measure is related to growth and waist circumference, independent of body weight, presenting the advantages of being low-cost, easy to obtain, and to interpret.^9^

 Simple anthropometric measurements used in the calculation of WHtR and CIndex have been studied for their performance in predicting body fat percentage and, consequently, associated cardiometabolic risks.^10^ However, such studies remain limited, preventing the establishment of consensus regarding pediatric cut-off points. 

 Given the implications of excess body fat in childhood and its impact later in life, this study aims to contribute to the literature on the applicability of anthropometric indicators in pediatric practice. Furthermore, it seeks to encourage their use in both individual and population-level contexts, expanding the possibilities for identifying childhood obesity beyond conventional diagnostic criteria. Accordingly, the objective of this study was to evaluate the performance of WHtR and CIndex as predictors of body fat percentage in children and to determine the most appropriate cut-off points for this population. 

## METHOD

 This is a cross-sectional, school-based, diagnostic test study, and used data from phase 1 of the "Nutrition and Health Study of Children and Adolescents in the City of Fortaleza — ENSCAFOR". The ENSCA-FOR study was approved by the Research Ethics Committee of the State University of Ceará (approval number 3.507.172/2019), in accordance with all ethical principles to ensure the safety and autonomy of participants, as established by Resolution 466/12. 

 Data collection was conducted in public schools under the jurisdiction of the Regional Executive Secretariats of Fortaleza, Ceará, between 2019 and 2023, accounting for the interruption caused by the COVID-19 pandemic. ENSCA-FOR assessed children and adolescents aged five to 17 years who were enrolled in municipal public schools. To estimate the total sample size, a 5% sampling error was considered, with a 95% confidence level and an expected prevalence of overweight of 50% to maximize the sample size, applying the formula for an infinite population. This yielded an estimated sample size of 1,483 children and adolescents. Further details on the sampling procedure can be found in a previous publication.^11^ Based on these data, all children evaluated in ENSCA-FOR aged five to nine years were included in the present study (n=314), representing 21.17% of the total sample. 

 Children of both sexes, aged five to nine years, who returned a signed informed consent form (ICF) provided by their parents and/or legal guardians participated in the study. Children with physical or cognitive disabilities that prevented participation in the data collection procedures were excluded. 

 Nutritional status was assessed through measurements of body weight, height, and waist circumference (WC). Body weight was measured using a portable electronic scale (Seca^®^), and height was measured using a portable stadiometer (Sanny^®^), ensuring standardized procedures. WC was measured using a non-elastic measuring tape (Sanny^®^). Body mass index (BMI) was calculated as weight divided by height squared (kg/m^2^) and used to assess nutritional status. Classification was based on BMI-for-age Z-scores using World Health Organization (WHO) growth reference curves, through the WHO AnthroPlus software (2007). 

 Body composition was assessed using BIA with a tetrapolar device (Biodynamics ^®^ model 450; 800 μA, 50 kHz). Body fat percentage (%BF) was classified according to the criteria proposed by Lohman et al. to determine excess body fat. A cutoff point of 20% was used for boys and 25% for girls. Values above these thresholds were considered indicative of excess body fat.^12^

 The CIndex was calculated using waist circumference, body weight, and height, according to the formula proposed by Valdez:^8^


$$\mathrm{CIndex}=\frac{\mathrm{waist}\;(m)}{0.109\times \sqrt{\mathrm{weight}\;(\mathrm{kg})/\mathrm{height}\;(m)}}$$

WHtR was obtained by dividing waist circumference (cm) by height (cm), following the methodology described by Ho et al.^13^

 For data analysis, numerical variables were tested for the nature of their distributions using the Kolmogorov-Smirnov test, and homogeneity was assessed using Levene’s test. Numerical data were expressed as means and standard deviations, while categorical variables were described using frequencies and percentages. 

 The means between the variables according to gender were compared using Student’s t-test for independent samples or the Mann-Whitney U-test, depending on the distribution of the data. The association between categorical variables and gender was assessed using Pearson’s χ^2^ test. 

 The relationship between the indices and excess body fat was assessed using the Mann-Whitney U-test, and the results were presented in graph form. 

 To assess the predictive power of the CIndex and WHtR in diagnosing excess body fat, Receiver Operating Characteristic (ROC) curve analysis was performed. The predictive power was tested using the area under the curve (AUC), its 95% confidence intervals (CI), sensitivity, and specificity, with body fat classification obtained by BIA serving as the gold standard. The cut-off point for each index was also determined in the test. 

 The AUC values were classified according to Metz,^14^ where AUC values are considered: poor (between 0.5 and 0.6); fair (>0.6 to 0.7); good (>0.7 to 0.8); very good (>0.8 to 0.9); and excellent (>0.9). The difference between the AUCs of CIndex and WHtR was tested using the Wald test and presented in graphical form. 

 The ROC curve analysis was performed using MedCalc^®^ software. Other analyses were conducted using the Statistical Package for the Social Sciences (SPSS) version 22.0. Statistical significance was considered at p<0.05. 

## RESULTS

 A total of 314 children were analyzed, the majority being female (56.7%), with an average age of 8.13 years (standard deviation — SD 1.06). The prevalences of overweight and obesity were 19.4 and 15.3%, respectively, while excess body fat was identified in 41.6% of the assessed children. A significant difference in the presence of excess body fat between sexes was also observed, with boys showing a prevalence of 56.2% compared to 43.8% in girls (p<0.001) (Table 1). 

**Table 1:** Anthropometric variables and body composition of children according to sex.

		Total	Females	Males	p-value
		n=314	n=178	n=136	
		mean (SD)	mean (SD)	mean (SD)	
Age (years) (n=500)		8.13 (1.06)	8.11 (1.07)	8.15 (1.05)	0.835^*^
Weight (kg)		31.23 (8.96)	31.14 (9.68)	31.35 (7.95)	0.341^*^
Height (m)		1.32 (0.08)	1.33 (0.09)	1.32 (0.08)	0.848^†^
BMI (kg/m^2^)		17.51 (3.38)	17.39 (3.54)	17.67 (3.17)	0.208^*^
Nutritional status, n (%)					
	Thinness	8 (2.5)	6 (75.0)	2 (25.0)	0.117
	Normal weight	197 (62.7)	117 (59.4)	80 (40.6)	
	Overweight	61 (19.4)	27 (44.3)	34 (55.7)	
	Obesity	48 (15.3)	28 (58.3)	20 (41.7)	
WtHR (cm)		0.45 (0.05)	0.45 (0.06)	0.46 (0.05)	**0.022** ^*^
WC (cm)		60.56 (9.46)	59.91 (9.96)	61.41 (8.71)	**0.065** ^*^
CIndex		1.15 (0.09)	1.13 (0.15)	1.17 (0.08)	**0.041** ^*^
%BF		21.62 (9.21)	21.56 (9.49)	21.67 (9.02)	0.935^*^
BF excess, n (%)					
	Absence	184 (58.6)	121 (65.8)	63 (34.2)	**<0.001**
	Presence	130 (41.4)	57 (43.8)	73 (56.2)	

Legend: SD: standard deviation; n: frequency; %: percentage; BMI: body mass index; WHtR: waist-to-height ratio; WC: waist circumference; CIndex: conicity index; BF: body fat; p-value:

* Mann-Whitney test;

† Student’s t-test for independent samples for numerical variables and Pearson’s χ^2^ test for categorical variables;

Bold indicates statistical significance: p<0.05.

**Figure 1:**
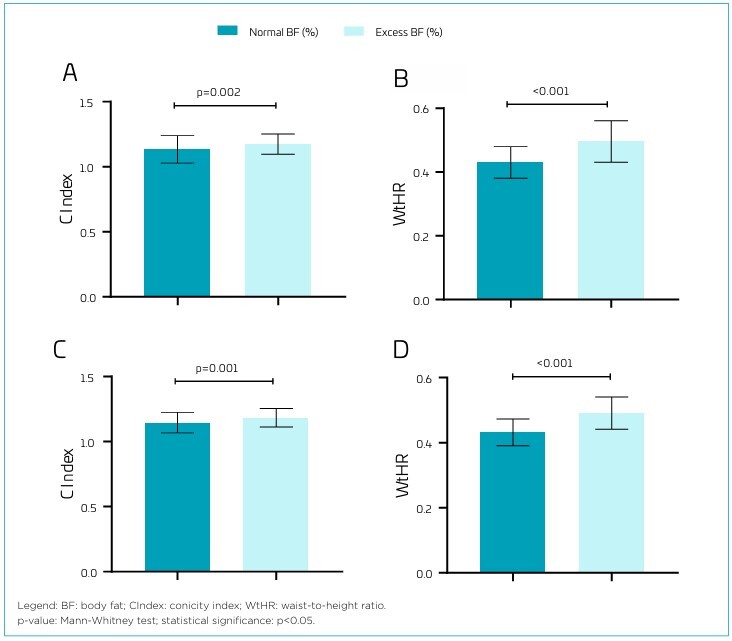
Comparison of mean values in the indices evaluated according to the presence of excess body fat in children, stratified by sex. **(A)** Conicity index in girls; **(B)** Waist-to-height ratio in girls; **(C)** Conicity index in boys; **(D)** Waist-to-height ratio in boys.

 The average values of WHtR and CIndex were significantly higher among children with excess body fat, regardless of sex (Figure 1). Boys with excess body fat had an average WHtR of 0.48 (SD=0.08) versus 0.43 (SD=0.04) in those without excess, while the average CIndex was 1.17 (SD=0.15) versus 1.14 (SD=0.08). Among girls, the average WHtR was 0.49 (SD=0.09) for those with excess body fat and 0.43 (SD=0.06) for those without, while the average CIndex was 1.15 (SD=0.17) and 1.12 (SD=0.15), respectively. 

Table 2 shows the performance analysis of the indices conducted through the area under the ROC curve. The WHtR was a good predictor of excess body fat in both sexes, with an AUC of 0.843 (95%CI 0.771–0.900) for boys and an AUC of 0.802 (95%CI 0.736–0.858) for girls, demonstrating specificity greater than 80% and sensitivity above 60%. To identify excess body fat in children, the optimal cut-off points based on the ROC curve for WHtR were >0.47 for boys and >0.45 for girls. In contrast, the CIndex showed an AUC of 0.652 (95%CI 0.565–0.731) for boys and an AUC of 0.647 (95%CI 0.572–0.717) for girls, being considered a poor predictor for excess body fat. 

**Table 2:** Area under the Receiver Operating Characteristics curve for determining the discriminatory power of waist-to-height ratio and conicity index in identifying excess body fat in children, according to sex.

		AUC (95%CI)	Sensitivity	Specificity	Cutoff	p-value
Males						
	WtHR	0.843 (0.771–0.900)	63.01	90.48	>0.47	<0.001
	CIndex	0.652 (0.565–0.731)	47.95	74.6	>1.17	0.001
Females						
	WtHR	0.802 (0.736–0.858)	73.68	80.17	>0.45	<0.001
	CIndex	0.647 (0.572–0.717)	54.39	72.73	>1.16	0.001

Legend: AUC: area under the curve; CI: confidence interval; WHtR: waist-to-height ratio; CIndex: conicity index. Gold standard: body fat percentage measured by bioelectrical impedance analysis; p-value: receiver operating characteristic curve. Statistical significance: p<0.05.

**Figure 2:**
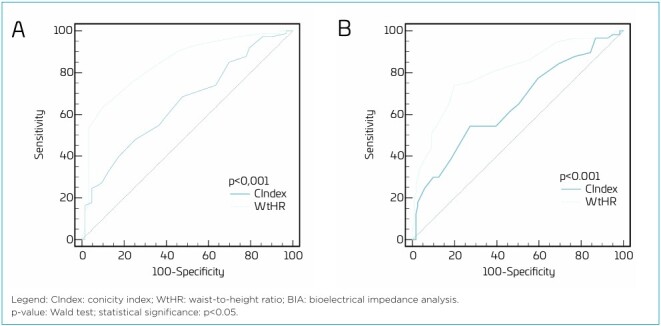
Comparison of the predictive power of WHtR and CIndex in identifying excess body fat assessed by BIA. **(A)** Males; **(B)** Females.

Figure 2 shows that, in the comparative analysis between the anthropometric indices, the performance of WHtR was significantly superior to that of the CIndex (p<0.001) in identifying excess body fat in children. 

## DISCUSSION

 Our study showed that WHtR yielded good results as a predictor of excess body fat in the children assessed, according to the AUC classification, while the CIndex performed considerably worse. It was also possible to confirm the superiority of WHtR over CIndex through a comparison of the two indices in the ROC curve. 

 The AUC values obtained for WHtR in our study indicate good discrimination capacity, consistent with the interpretation criteria established for diagnostic tests. Although there is no absolute consensus on the ideal cut-off values, sensitivity above 60% combined with a specificity of over 80% is generally considered acceptable for screening purposes, especially in population-based studies using simple, low-cost anthropometric markers, especially with an AUC greater than 0.8.^15^

 The WHtR cutoff points appear to be applicable in both clinical and research settings for identifying excess body fat in the pediatric population. Furthermore, values exceeding these cutoffs may indicate potential cardiometabolic risk. Several methods have been studied and used as screening tools for obesity, excess body fat, and related comorbidities across all age groups. 

 However, some indices have important limitations, such as the BMI, which does not distinguish between lean mass and fat mass. In our study, BMI identified a prevalence of overweight that was twice as low as the prevalence of excess body fat, reinforcing that, while BMI has high specificity for diagnosing obesity, it shows only moderate sensitivity for detecting excessive adiposity. In this context, WHtR has gained prominence as a safe and effective anthropometric index for predicting body fat percentage in children, as demonstrated in our findings. In addition to its effectiveness, it offers a simple calculation based on easily obtainable measures.^16,17^

 WHtR proportionally reflects the distribution of body fat, especially central fat, which is associated with an increased cardiometabolic risk. This ratio differs from other indices because it is not influenced by body weight, as waist circumference is adjusted for height, allowing for a more accurate assessment of abdominal fat accumulation relative to body size. Due to these proportions, WHtR becomes a sensitive tool for identifying individuals with a higher predisposition to developing outcomes that exacerbate cardiovascular risks, even in the absence of obesity.^18^

 A meta-analysis of eight studies concluded that WHtR can predict central obesity with high accuracy in children and adolescents of various races, ages, and regardless of sex, including the Brazilian population, providing a universal cut-off point of 0.49,^19^ a value close to that found in our study. However, since WHtR is a tool used for both screening and diagnostic purposes, it is essential that it demonstrates adequate sensitivity — that is, the ability to correctly identify children with excess body fat. The cut-off points proposed in our study showed higher sensitivity compared to the universal cut-off for boys (39.73%) and girls (43.86%), which contributes to reducing the risk of false negatives. Although the universal cut-off point presented higher specificity for boys (96.83%) and girls (90.91%), it is important to emphasize that, within the context of screening, sensitivity is the most relevant parameter, as specificity refers to the ability of the tool to correctly identify individuals without excess body fat. Therefore, in primary health care settings, where early detection and prevention are key priorities, sensitivity is often valued over specificity. 

 Considering the diagnosis of excess body fat, sex-stratified cutoff points are necessary due to physiological, hormonal, and sexual maturation differences between boys and girls. These cutoff points should take into account the distinct patterns of fat distribution between sexes, as boys generally exhibit lower fat accumulation compared to girls and tend to have a central fat distribution pattern, concentrated around the abdomen, whereas girls typically present a peripheral distribution, mainly in the hips and thighs.^20,21^ Our findings also demonstrate disparities in body fat accumulation between sexes; however, we observed a higher prevalence of excess body fat among boys compared to girls, which may be explained by specific environmental and behavioral characteristics of the studied population, as well as divergences in the analytical and classification tools used.^22^

 Another study, conducted from a birth cohort in the United Kingdom, presented interesting aspects that may help in understanding the good performance of WHtR in predicting excess body fat. WHtR showed a high correlation with fat mass and a low correlation with lean mass in children throughout the follow-up. Furthermore, WHtR remained relatively unchanged in children with normal growth into young adulthood, highlighting the stability of WHtR regardless of sex and age.^23^

 The efficacy of WHtR is demonstrated by Dou et al.,^24^ with precision in identifying children and adolescents with high cardiometabolic risk (CMR), even in seemingly healthy populations. The use of appropriate cut-off points allows WHtR to demonstrate sensitivity in groups affected by the combination of three or more CMR factors, reiterating its value as a predictive tool. Additionally, another study points to this relationship as a highly sensitive index in identifying CMR factors such as diabetes, hypertension, dyslipidemia, and coronary artery disease in adults.^25^

 These results can be justified by the fact that abdominal fat is closely related to cardiometabolic disorders. Its presence may indicate a high concentration of apolipoprotein B (ApoB), the protein component of atherogenic lipoproteins, particularly in LDL, which is an important biochemical marker for atherosclerosis, even at younger ages. The lipoproteins VLDL, IDL, and LDL contain ApoB-100, which facilitates the fluidity and transport of these lipoproteins through plasma, promoting their adhesion to the endothelium.^26^

 This process of atherogenesis is directly related to risk factors for cardiovascular diseases (CVD). Many of these factors, such as obesity, excess abdominal fat, insulin resistance, and dyslipidemia, begin in childhood and persist into adulthood, increasing the likelihood of cardiovascular injuries or worsening already existing conditions. The progression and severity of atherosclerosis will depend on how these risk factors interact and how they are managed. Therefore, early screening and interventions are ways to prevent adverse events later.^27^

 The CIndex was proposed by Valdez,^8^ with the objective of identifying excess body fat in adults, based on the resemblance of the body to a double cone or cylinder in relation to abdominal fat excess, using the anthropometric markers of weight, height, and WC. This index is an important tool for assessing the distribution of body fat and the cardiometabolic risks associated with it in adults. However, studies with other populations still need to be developed to validate its effectiveness and determine its possible cut-off points. 

 For the CIndex, the cut-off points found in our study did not show good sensitivity and specificity, and the AUC showed low values. Sant’Anna et al.^28^ conducted research with individuals in the same age range as ours and found that the AUC for the CIndex in males was 0.719 (95%CI 0.621–0.804), while for females the result was 0.762 (95%CI 0.668–0.840), with statistical significance (p<0.001) in both sexes. 

 The prediction of android fat in children was measured by Filgueiras and collaborators^29^ through CIndex, WC, and WHtR, finding similar AUC values between the three indices; however, the sensitivity of the CIndex was lower. Furthermore, android fat is characterized as fat located in the region between the ribs and pelvis, which may have contributed to the good performance of the CIndex in that study and differs from our study, which assessed total body fat. 

 The lower performance of the CIndex in predicting CMR in children can be attributed to the physiological and methodological limitations of the indicator, as it was developed for another population. By incorporating total body weight into the equation, the index becomes sensitive to variations in total body mass, reducing its specificity for adipose tissue. In children, this limitation is further intensified by natural growth changes, such as increases in bone mass, muscle mass, and hydration. Compared to other indicators, including WHtR, a study that followed adolescents for three years concluded that the CIndex showed inferior predictive performance and was only effective in the third measurement.^30^

 This study has some limitations that should be considered when interpreting the results, including the use of BIA as the assessment method. Although BIA is widely reported in the literature as a feasible and practical technique and shows good correlation with other methods, it is not considered the gold standard for this analysis, especially in children. Compared to DEXA, BIA may overestimate fat mass and body fat percentage, particularly in children and adolescents with higher adiposity, exhibiting reduced accuracy and an increased risk of measurement errors in these participants.^31^ However, BIA is a more practical method for assessing the pediatric population because it is quick, whereas DEXA requires the child to remain still for 10 to 20 minutes. 

 In addition, our sample is not representative of the Brazilian population; however, the internal comparisons made between the groups allow valid inferences to be made about the performance of the anthropometric indicator assessed. It is important to point out that generalization of our findings should be done with caution, considering that the study was made up of schoolchildren from a specific region of the country, and factors such as color/race and ethnicity can influence the distribution of body fat and anthropometric parameters, impacting the performance of indices such as WHtR, which also limits generalization of our results to populations of different ages. Although other studies have investigated the applicability of the WHtR in adolescents, our results should not be extrapolated directly to this age group without further investigation. Despite these limitations, we emphasize the representativeness of the sample in the local context and encourage further studies with more diverse and representative samples to verify the applicability of the cut-off points in different population contexts. 

 Our study helps to fill a gap in the current literature, given the scarcity of research focused on predicting excess body fat in children, especially in the age group we worked with. The findings reinforce the good performance of the WHtR as a viable tool for estimating body fat percentage, encouraging its applicability in both clinical practice and research. As a simple, low-cost method with good reproducibility, it facilitates its incorporation into settings with limited resources and expands its use in early CMR screening in childhood. 

 We suggest that longitudinal follow-up studies will be necessary to measure the performance of the indices throughout child development. Based on these results, it will be possible to contribute to the early diagnosis of outcomes secondary to adiposity, allowing for greater surveillance and research, and providing appropriate interventions to minimize the impacts of childhood obesity in adulthood. 

 Based on our findings, it was possible to conclude that the WHtR is a good predictor of excess body fat in children of both sexes, while the CIndex demonstrated a considerably inferior performance. Including the measurement of WHtR in pediatric health surveillance can provide additional information not only related to body weight but also to the risk of developing cardiometabolic complications throughout life. Further studies are needed to identify reliable cutoff points in the pediatric population at the national level, with the aim of establishing a consensus on the future use of this index. 

## Data Availability

The database that originated the article is available with the corresponding author.
